# Role of Dual-Energy Computed Tomography in the Identification of Monosodium Urate Deposition in Gout Patients: A Comprehensive Analysis of 828 Joints According to Structural Joint Damage

**DOI:** 10.7759/cureus.19930

**Published:** 2021-11-27

**Authors:** Le Huu Hanh Nhi, Le Huu Nhat Minh, Thuan Minh Tieu, Esraa Mahmoud Mostafa, Sedighe Karimzadeh, Nguyen Minh Dung, Nguyen Hai Nam, Le Van Phuoc, Nguyen Tien Huy

**Affiliations:** 1 Department of Radiology, Vinmec Healthcare System, Ho Chi Minh City, VNM; 2 Faculty of Medicine, University of Medicine and Pharmacy at Ho Chi Minh City, Ho Chi Minh City, VNM; 3 Department of Medicine, McMaster University, Hamilton, CAN; 4 Department of Medicine, Port Said University, Port Said, EGY; 5 Online Research Club, School of Tropical Medicine and Global Health, Nagasaki University, Nagasaki, JPN; 6 Department of Medicine, Vietnam National University, Ho Chi Minh City, VNM; 7 Department of Surgery, Kyoto University, Kyoto, JPN; 8 Department of Radiology, Cho Ray Hospital, Ho Chi Minh City, VNM; 9 School of Tropical Medicine and Global Health, Nagasaki University, Nagasaki, JPN

**Keywords:** monosodium urate deposition, urate, dect, dual-energy computed tomography, gout, radiography, musculoskeletal imaging

## Abstract

Background

Dual-energy computed tomography (DECT) has become a promising, non-invasive procedure for the visualization, characterization, and quantification of monosodium urate (MSU) crystals, which aids clinicians in the diagnosis of gout. In this study, we aimed to examine the diagnostic accuracy of DECT in the evaluation of gout.

Methodology

This cross-sectional retrospective study included patients who were clinically diagnosed with gout and underwent a DECT scan.

Results

A majority (80.4%) of the MSU deposits were found in the ankle joints. The presence of MSU deposits on DECT scan was highly correlated with bone erosion in the upper limb (odds ratio [OR] = 132; 95% confidence interval [CI] = 17.3-1004.3), bone sclerosis in the lower limb (OR = 36.4; 95% CI = 15.4-86.1), bone erosion in metacarpophalangeal joints (OR = 160; 95% CI = 42.7-600.2), and bone sclerosis in metatarsophalangeal joints (OR = 35.6; 95% CI = 15.5-81.9). Using linear regression analysis on patient-level data, correlations were found between DECT MSU crystal deposition and damage on all categories of structural joint damage showing significant association with erosion (r = 0.91, p < 0.001) and space narrowing (r = 0.75, p < 0.001) but not with joints having periarticular calcification (r = 0.52, p < 0.041).

Conclusions

Our study established DECT as a valid method for detecting MSU deposits and their association with structural joint deterioration in a Vietnamese population.

## Introduction

Gout is a common form of inflammatory arthritis that is characterized by elevated serum uric acid and deposition of monosodium urate (MSU) crystals in the joints and soft tissue [1]. The long-term clinical consequences of MSU deposition in joints are tophi, which can present in acute or chronic forms. In its acute form, gouty arthritis is characterized by sudden-onset intense and recurring pain [2]. Hence, the urgent detection of MSU is crucial for diagnosis and management. Microscopic analysis of synovial fluid looking for needle-shaped MSU crystals by arthrocentesis is the gold standard tool for the diagnosis of gout [3]. Other medical conditions such as rheumatoid arthritis, osteoarthritis, infectious arthritis, or calcium pyrophosphate deposition share similar clinical presentations as gout. While serum uric acid concentration and medical imaging such as X-ray and ultrasound assist in the diagnosis, the gold standard for establishing a definite diagnosis of gout is the presence of MSU in aspirated joint fluid [4]. Several diagnostic methods have been developed for gout diagnosis. The clinical features of gout such as intense pain with tenderness may be absent in some patients, some patients may show a normal serum uric acid level, and sometimes uric acid crystals cannot be detected using the standard methods. In these circumstances when a definite diagnosis cannot be established, imaging modality can help in the identification of affected joints and their burden.

Imaging modalities such as ultrasonography (US), magnetic resonance imaging (MRI), conventional computed tomography (CT), and dual-energy computed tomography (DECT) can help in the identification of clinical and intra-articular tophi [5]. DECT scan has been suggested for the diagnosis of gout in the 2015 Gout Classification Criteria, an American College of Rheumatology/European League Against Rheumatism Collaborative Initiative. DECT is a non-invasive test with high sensitivity and specificity to detect MSU crystals in affected joints, tendons, ligamentous, and involved tissues [5]. The technique helps detect small-sized crystals in uncommon areas before clinical manifestation, assisting clinicians in the diagnosis as well as treatment. On the other hand, arthrocentesis, or joint aspiration, is an invasive procedure and can be difficult to perform on an acute inflammatory small joint; moreover, it carries the risk of bleeding, infection, or damage to nearby structures [6]. DECT can detect four times more tophi than clinical examination alone [7].

Although many studies have been conducted worldwide [8-10] using DECT for the diagnosis of gout, it is not commonly performed in Vietnam. Although a few centers are capable of performing this technique, it is not widely employed to date and no publications exist. Therefore, we conducted this study to evaluate a new technique for diagnosing gout in clinical practice.

## Materials and methods

Study population

A cross-sectional study was performed on 36 patients diagnosed with gout at Cho Ray Hospital, Ho Chi Minh City, Vietnam from March 2017 to May 2018. All patients were diagnosed with gout using clinical findings, serum uric acid testing, and imaging methods with scores >8 based on the American College of Rheumatology/European League Against Rheumatism 2015 guidelines. Patients who underwent DECT in the lower and upper extremities were included. Patients whose imaging had technical errors when DECT was employed or those who had artifacts in DECT imaging were excluded. Because only recorded findings and stored images were collected, there was no interference with patients’ or clinicians’ treatment decisions. The personal information of the patients involved in the study was confidential. The study was approved by the Ethics Research Council of the University of Medicine and Pharmacy at Ho Chi Minh City, Vietnam.

Study protocol

Clinical assessment, classification, and DECT scanning were performed from March 2017 to May 2018 for patients diagnosed with gout. Collected data included demographic characteristics, MSU crystal deposition, and blood uric acid findings of patients based on medical records (inpatient and outpatient).

Dual-energy computed tomography protocol

DECT scanning was performed using SOMATOM Definition Edge (Siemens Medical, Erlangen, Germany) with a filter in front of the tube and no contrast injections. The scanning position was the patient lying on the back with arms facing towards the head. The scanning area was from above the elbow joint at least 5 cm to the end of the finger and from above the knee joint at least 5 cm to the end of the toes. The scanner started shooting at 120 kVp, and then two sets of data were developed using a gold filter plate with a low energy spectrum (80 kVp) and tin with a high energy spectrum (140 kVp). Based on the calculations of three-dimensional images and predetermined thresholds, sodium urate crystals and bone were color-coded for easier visualization and volume calculation. Two-dimensional and three-dimensional color maps were recorded and integrated with corresponding CT images to simultaneously display the anatomical structure and urate crystal deposition location. The imaging features of structural joint damage that were recorded included MSU deposition, bone erosion, periarticular calcification, joint space narrowing, and bone sclerosis. After collection, data were transferred into the Syngovia system. The DECT images of patients were processed and three-dimensionally rendered by supporting software.

Statistical analysis

Data were input into Microsoft Excel and analyzed using SPPS version 22.0 (IBM Corp., Armonk, NY, USA). Categorical variables were presented as proportions while the median (range) or mean (standard deviation) were used to summarize continuous variables, which included the clinical and imaging characteristics of our samples. Pearson’s correlation tests were used to investigate correlations between variables. Linear regression models were used to examine the relationship between DECT MSU crystal deposition and serum urate concentration on joint damage. The tests were considered statistically significant at p-values of <0.05. The correlation coefficient (r) and standardized beta (β) were defined as follows: -1 indicates a strong negative correlation, 0 indicates no association, and +1 indicates a strong positive correlation.

## Results

Patient characteristics

**Figure 1:**
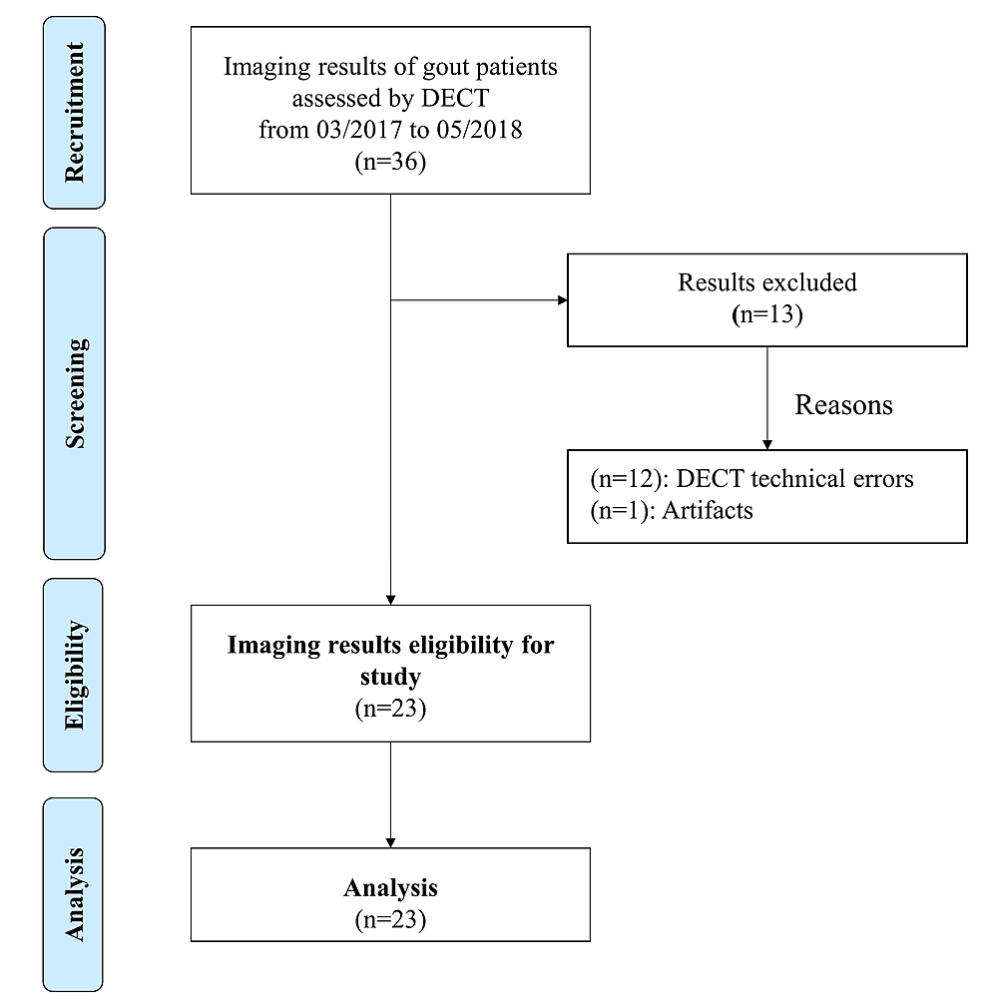
Flow diagram of patient enrollment. DECT: dual-energy computed tomography

The DECT results of 23 patients were selected for analysis in this study (Figure 1). All participants were males, with the mean age and standard deviation of 55.3 and 13.8 years. The median (interquartile range) disease duration was 7.5 (4-10) years. Subcutaneous tophus was noted in 20 (87%) patients. There were 14 (61%) patients with gout disease for more than three years. The mean and standard deviation of serum uric acid level was 8.3 and 1.7 mg/dL, respectively. Further, among these patients, the median (interquartile range) volume of overall urate deposition was 8.88 (1.18-15.40) cm^3^.

Clinical characteristics

**Figure 2:**
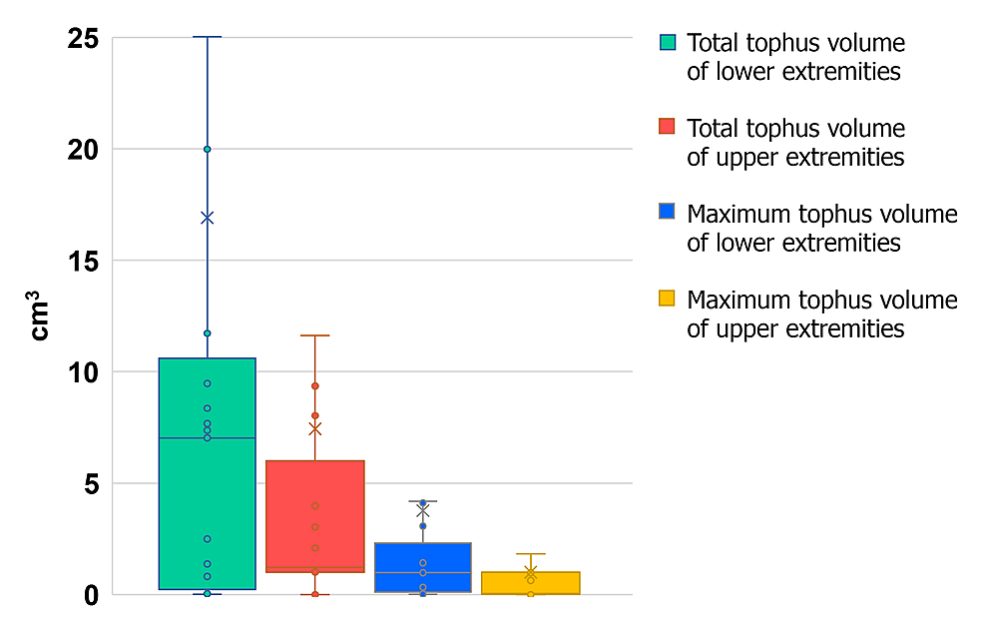
Distribution of tophi volume by extremity.

Regarding the distribution of tophi volume, 75% of the patients had a total tophi volume of approximately 10.5 cm^3^ or lower and approximately 6 cm^3^ or lower in the lower and upper extremities, respectively. Overall, 75% of the patients had the maximum tophi volume of 2.3 cm^3^ in the lower extremities and approximately 1 cm^3^ or lower in the upper extremities (Figure 2).

The distribution according to the general area affected in the lower and upper extremities is shown in Table 1. There were 104 (56.5%; 95% confidence interval [CI] = 49.3%-63.7%) and 63 (34.2%; 95% CI = 27.4%-41.1%) joints with DECT urate deposition in lower and upper limbs, respectively. Sclerosis was the most commonly observed structural joint damage, with 113 joints (61.4%; 95% CI = 54.4%-68.5%) in the lower limbs and 44 joints (23.9%; 95% CI = 17.7%-30.1%) in the upper limbs. In comparison, space narrowing was the least observed feature, with 59 (32.1%; 95% CI = 25.3%-38.8%) and 28 (15.2%; 95% CI = 10%-20.4%) joints in lower and upper limbs, respectively.

**Table 1:** Distribution according to the general area affected in lower and upper extremities. CI: confidence interval

Imaging feature	Monosodium urate crystals, % (95% CI)	Bone erosion, % (95% CI)	Periarticular calcification, % (95% CI)	Joint space narrowing, % (95% CI)	Bone sclerosis, % (95% CI)
Lower extremities (n = 46)					
Knee	71.7% (58.6-84.9)	32.6% (18.9-46.3)	63% (48.9-77.1)	37% (22.9-51.1)	84.8% (74.3-95.3)
Ankle joints	80.4% (68.8-92)	43.5% (29-58)	69.6% (56.1-83)	28.3% (15.1-41.4)	78.3% (66.2-90.3)
Metatarsophalangeal joints	45.7% (31.1-60.2)	47.8% (33.2-62.4)	45.7% (31.1-60.2)	34.8% (20.9-48.7)	47.8% (33.2-62.4)
Interphalangeal joint	28.3% (15.1-41.4)	32.6% (18.9-46.3)	28.3% (15.1-41.4)	28.3% (15.1-41.4)	34.8% (20.9-48.7)
Total affected joints	56.5% (49.3-63.7)	39.1% (25-53.3)	51.6% (44.4-58.9)	32.1% (25.3-38.8)	61. 4% (54.4-68.5)
Patients affected lower extremities (%)	100	52.2	82.6	56.5	91.3
Upper extremities (n = 46)					
Elbow	58.7% (44.3-73.1)	21.7% (9.7-33.8)	28.3% (15.1-41.4)	17.4% (6.3-28.5)	17.4% (6.3-28.5)
Carpometacarpal joint	34.8% (20.9-48.7)	19.6% (8-31.2)	21.7% (9.7-33.8)	17.4% (6.3-28.5)	45.7% (31.1-60.2)
Metacarpophalangeal joint	23.9% (11.5-36.4)	23.9% (11.5-36.4)	19.6% (8-31.2)	15.2% (4.7-25.7)	21.7% (9.7-33.8)
Interphalangeal joint	19.6% (8-31.2)	8.7% (0.5-16.9)	10.9% (1.8-20)	10.9% (1.8-20)	10.9% (1.8-20)
Total affected joints	34.2% (27.4-411)	18.5% (12.9-24,1)	20.1% (14.3-25.9)	15.2% (10-20.4)	23.9% (17.7-30.1)
Patients affected in upper extremities (%)	82.6	26.1	52.2	26.1	21.7

A total of 828 joint sites were assessed to determine the relationship of MSU deposition in DECT with structural joint damage on plain CT. The sites are divided into the following four areas: upper limb, lower limb, metacarpophalangeal (MCP), and metatarsophalangeal (MTP) (Table 2).

**Table 2:** Relationship of DECT MSU crystal deposition with structural joint damage on plain CT: site-by-site analysis according to the general area. ^#^logistic regression was used to calculate odds ratios and 95% confidence intervals. CD: crystal deposition; DECT: dual-energy computed tomography; MSU: monosodium urate; CT: computed tomography

Erosion present	Erosion absent	Periarticular calcification present	Periarticular calcification absent	Joint space narrowing present	Joint space narrowing absent	Bone sclerosis present	Bone sclerosis absent	
Upper extremities (n = 184)								
33	30	34	29	28	35	37	26	
1	120	3	118	0	121	7	114	
132 (17.3-1004.3)^#^		46.1 (13.2-160.7)^#^		NA		23.2 (9.3-57.8)^#^		
Lower extremities (n = 184)								
65	39	80	24	53	51	95	9	
7	73	15	65	6	74	18	62	
17.4 (7.3-41.5)^#^		14.4 (7-29.8)^#^		12.8 (5.1-32)^#^		36.4 (15.4-86.1)^#^		
Metacarpophalangeal (n = 230)								
20	5	20	5	15	10	22	3	
5	200	8	197	0	205	11	194	
160 (42.7-600.2)^#^		98.5 (29.4-329.8)^#^		NA		129.3 (33.5-499.2)^#^		
Metatarsophalangeal (n = 230)								
62	16	41	37	40	38	70	8	
16	136	20	132	9	143	30	122	
32.9 (15.5-70.1)^#^		7.3 (3.8-14.0)^#^		16.7 (7.5-37.5)^#^		35.6 (15.5-81.9)^#^		

Upper Limb Joint Sites

In total, 184 joint sites showed MSU deposition on DECT. In DECT findings, MSU deposition sites associated with bone erosion and periarticular calcification were statistically significantly correlated with sites that did not have MSU deposition (odds ratio [OR] = 132; 95% CI = 17.3-1004.3, and OR = 46.1; 95% CI = 13.2-160.7, respectively; p < 0.001). Additionally, bone sclerosis had a statistically significant relationship with DECT (p < 0.001) but did not vary considerably (OR = 23.2; 95% CI = 9.3-57.8) (Table 2).

Lower Limb Joint Sites

The number of sites was the same as the upper limb (n = 184 sites). As expected, MSU deposition sites on DECT were significantly associated with a greater risk of bone sclerosis, bone erosion, periarticular calcification, and joint space narrowing compared with missing MSU deposition sites (OR = 36.4, 95% CI = 15.4-86.1; OR = 17.4, 95% CI = 7.3-41.5; OR = 14.4, 95% CI = 7.0-29.8; OR = 12.8, 95% CI = 5.1-32, respectively; p < 0.001) in a descending order (Table 2).

Metacarpophalangeal Joint Sites

In 230 sites tested by DECT, compared with absent MSU deposition sites, sites with MSU deposition were as likely to have joint injury in the following three features: bone erosion (OR = 160; 95% CI = 42.7-600.2), bone sclerosis (OR = 129.3; 95% CI = 33.5-499), and periarticular calcification (OR = 98.5; 95% CI = 29.4-329.8) (p < 0.001) (Table 2).

Metatarsophalangeal Joint Sites

Similarly, 230 MTP sites assessed by DECT also highlighted the relationship between MSU deposition sites and joint injuries on plain CT. MSU deposition sites on DECT showed an increased risk of bone sclerosis, bone erosion, and periarticular calcification compared with absent MSU deposition sites (OR = 35.6, 95% CI = 15.5-81.9; OR = 32.9, 95% CI = 15.5-70.1; OR = 7.3, 95% CI = 3.8-14, respectively; p < 0.001) (Table 2). Using linear regression analysis on patient-level data, significant correlations were found between DECT MSU crystal deposition and damage on all categories of structural joint damage (p < 0.05 for erosion, calcification, space narrowing) but not sclerosis (p = 0.29).

The opposite finding was noted in the case of serum urate concentration (mg/dL), which had no statistically significant relationship with any features of structural joint damage (p > 0.05 for all categories). Positive correlations were observed between DECT MSU crystal deposition and serum urate concentration regarding erosion, calcification, and space narrowing of the joint (p < 0.001, p < 0.041, p < 0.001, respectively). However, no statistically significant relationship was noted between these two predictors in sclerosis (p < 0.12) (Table 3). A strong linear relationship was observed in the frequency of MSU crystal deposition with erosion.

**Table 3:** Linear regression analysis showing the relationships between DECT MSU crystal deposition and serum urate with structural joint damage on plain CT: patient-level analysis (n = 23). DECT: dual-energy computed tomography; MSU: monosodium urate

Dependent variable	Predictors	β (SE)	Standardized β	P-value	Model (r, R^2^, F, p-value)
Number of joints with erosion	Number of joints with DECT MSU crystal deposition	0.99 (0.1)	0.92	<0.001	r = 0.91, R^2^ = 0.83, F = 48.4, p < 0.001
	Serum urate concentration (mg/dL)	-0.09 (0.22)	-0.04	0.7	
Number of joints with periarticular calcification	Number of joints with DECT MSU crystal deposition	0.48 (0.18)	0.53	0.01	r = 0.52, R^2^ = 0.27, F = 3.8, p < 0.041
	Serum urate concentration (mg/dL)	-0.09 (0.38)	-0.05	0.81	
Number of joints with joint space narrowing	Number of joints with DECT MSU crystal deposition	0.67 (0.14)	0.76	<0.001	r = 0.75, R^2^ = 0.56, F = 12.6, p < 0.001
	Serum urate concentration (mg/dL)	-0.12 (0.29)	-0.07	0.67	
Number of joints with sclerosis	Number of joints with DECT MSU crystal deposition	0.29 (0.27)	0.22	0.29	r = 0.44, R^2^ = 0.19, F = 2.4, p < 0.12

## Discussion

From March 2017 to May 2018, we enrolled 23 eligible patients in this study. MSU deposits were mostly found in the ankle joints. The presence of MSU deposits detected on the DECT scan was highly correlated with bone erosion in the upper limb and MCP joints and with bone sclerosis in the lower limb and MTP joints. All included patients were males, which is expected as gout is predominantly seen in males [11]. In addition, we excluded female participants with gout. Hence, in our study, the male-to-female ratio is higher than that reported in other similar studies [12-14]. The mean age in our study was 55 ± 14 years, ranging from 37 to 88 years. The mean age reported by Choi et al. (2012) [12], Mallinson et al. (2013) [13], and Dalbeth et al. (2014) [14] was 62, 61.3, and 58, respectively. According to the literature, gout is not common in patients under the age of 45, but 26.1% (n = 6/23) of our participants were under the age of 45. This suggests an increasing incidence of gout in the younger population. Hyperuricemia occurs when the serum uric acid level is over 7 mg/dL in males and 6 mg/dL in females. The condition can be caused by increased production of uric acid, a decrease in the renal secretion of uric acid, or a combination of these causes. The mean serum uric acid level in our study was 8.3 ± 1.7 mg/dL, which is higher than similar studies by Choi et al. [12] (6.3 mg/dL) and Dalbeth et al. [14] (7.2 and 2.0) mg/dL). Of these, 91.3% (n = 21/23) of our participants had serum uric acid levels above 6 mg/dL, which is significantly higher compared to other studies. Hyperuricemia is an important risk factor for incident gout [15]. However, Schlesinger et al. [16] found no correlation between the incidence of gout and serum uric acid level. Of 339 patients, one-third with acute gout had a serum uric acid level lower than 8 mg/dL [16]. In another study, the risk of gout increased from 0.33% in patients with serum uric acid levels less than 6 mg/dL to 26% in patients with serum uric acid levels more than 10 mg/dL in five years. The 15-year cumulative incidence ranged from 1.1% for <6 mg/dL to 49% for >10 mg/dL [17]. On one hand, we found MSU crystal disposition in asymptomatic joints in patients with hyperuricemia. On the other hand, there were cases where even though hyperuricemia was confirmed and the joints showed clinical symptoms, MSU crystal disposition was not found on the DECT scan. Therefore, maintaining normal serum uric acid levels is the goal in gout management. A serum uric acid level below 6 mg/dL helps dissolve MSU crystals, thus reducing the clinical symptoms [18,19].

**Figure 3:**
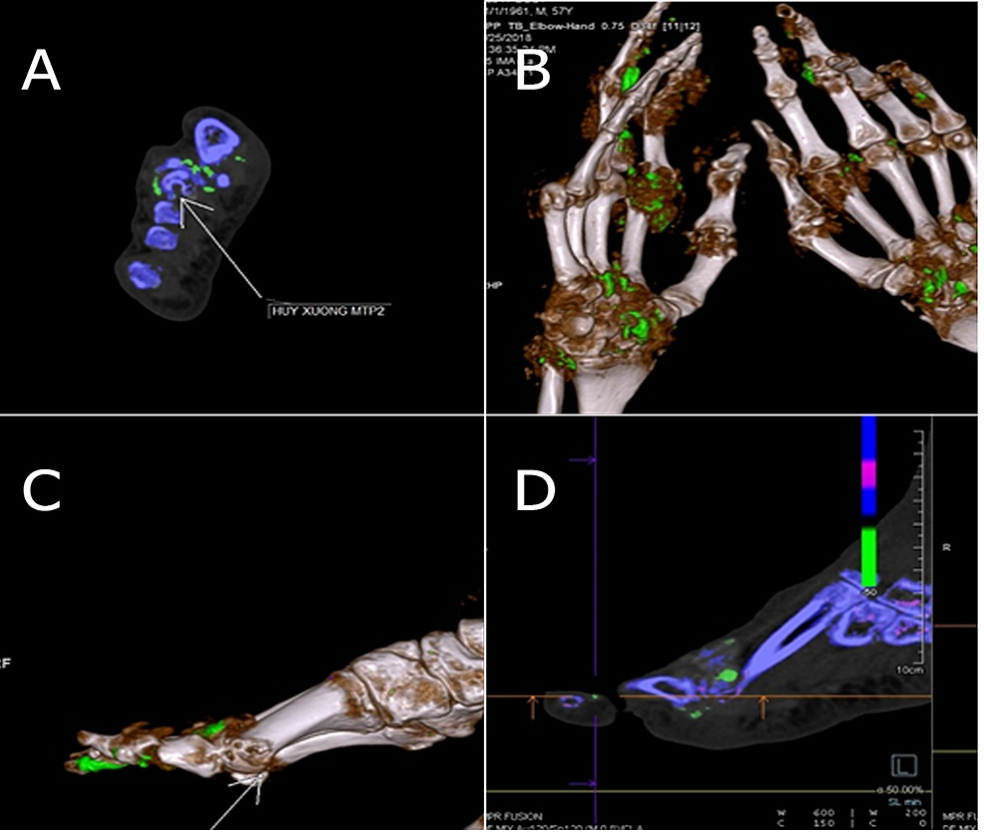
MSU crystal deposition and periarticular calcification. A 67-year-old male was diagnosed with gout and presented with hyperuricemia (10 mg/dL). DECT images illustrated the sites of MSU crystal deposition; green color for MSU inside and outside of joints, and blue for periarticular calcification. DECT: dual-energy computed tomography

In this study, 87% (20/23) of the patients presented with clear clinical symptoms. We observed MSU crystal depositions more frequently and with a larger volume in gout patients with more obvious symptoms. In addition, there were cases where MSU crystal depositions were detected in asymptomatic patients with low serum uric acid levels. This can be explained by the large number of participants who were on medications for hyperuricemia in this study (70%). However, this percentage is lower than that reported in other similar studies: Choi et al. [12] (87%) and Dalbeth et al. [14] (92%). In addition, 60.9% (14/23) of the participants had chronic gout for more than three years, with higher MSU crystal deposition volume than newly diagnosed gout patients. Regarding lower limb damage, 90% of first joint damage in patients with gout occurred in a single joint [20]. MSU crystal deposition can be seen inside and surrounding the joint in Figure 3.

Gout most commonly affects the MTP joint, as shown by clinical trials and imaging studies [21,22]. While all joints can be affected, lower limb joints are more often damaged than upper limb joints [21]. In our study, MSU crystals were detected in 48% (n = 179/368) of the areas in the lower limb, almost twice of those detected in the upper limb at 25.5% (n = 94/368).

**Figure 4:**
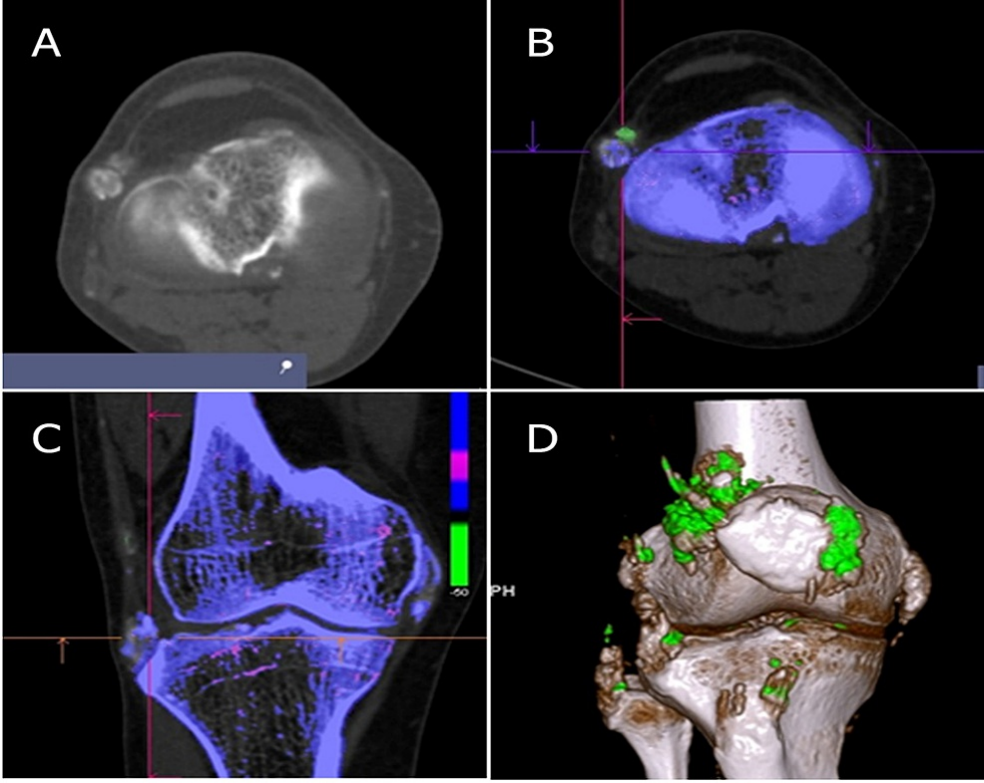
Structural damage in joints with MSU crystal deposition. A 57-year-old male was diagnosed with gout and presented with hyperuricemia. Urate is shown in green color. Bone erosion is shown at right MTP2 (crystal volume V = 1.53 cm^3^) (A) and periarticular calcification is shown at left MTP1 (D). DECT 3D images also illustrated deformities at right MCP5 (crystal volume V = 0.01 cm^3^) and right MTP1 (crystal volume V = 0.97 cm^3^) (B and C). DECT: dual-energy computed tomography; MSU: monosodium urate; 3D: three-dimensional; MTP: metatarsophalangeal; MCP: metacarpophalangeal

In previous studies, MSU crystals were often found in the tendons of patients with chronic gout [23]. In knee joints, the meniscus is prone to MSU crystals; in the ankle, it is the body and root of the Achilles tendon; in the foot, it is the first MTP joint. In this study, 56.5% (n = 104/184) of the joints had MSU crystal deposition, predominantly in the ankle. In the upper limb, the MCP2 was the most commonly affected, while MCP4 was the least affected. In this study, 33.9% (n = 78/230) of the MTP joints had MSU crystal deposition. In the lower limb, MTP1 was the most commonly affected, while MTP5 was the least affected, which is in accordance with the study by Dalbeth et al. [14]. There are several hypotheses to explain why MTP1 is the most commonly affected joint. When the big toe is bent toward other toes, the joint is bumped (bunion or hallux valgus). Gravitational force, when standing or walking, is exerted on the joints, causing pain. This joint deformity can also result from a weak foot structure due to genetics or other joint inflammatory conditions. In a recent study, the prevalence of bunion or hallux valgus was 41% in patients with gout compared to 25% in the control group [24]. MTP1 joint is the most prone to physical force and is located far away from the body at a low temperature. These are ideal conditions for MSU crystals to develop. In the upper limb, the triceps tendon was the most affected, which is consistent with the findings of Mallinson et al. In our study, the most affected areas with MSU were interracial, triceps tendon, and olecranon bursa, whereas in another study conducted by Mallinson et al., triceps tendon, hand tendons, and interracial tendons were the most affected regions [13]. In this study, 34.2% (n = 64/184) had MSU crystal deposition, predominantly in the elbow. MCP and MTP damage was also seen in joints with MSU crystal deposition (Figure 4). Overall, damage in the upper limb was less common than that in the lower limb.

Our study is constrained by the small sample size. Moreover, the nature of cross-sectional investigations did not allow us to follow the progression of the disease. Furthermore, DECT has not been compared to other modern communication technologies. Further longitudinal studies with bigger sample sizes are required to better understand the natural history of the disease, the repercussions of the disease, and to compare the disease with other diagnostic modalities.

## Conclusions

Our study demonstrated DECT as a reliable tool to detect MSU deposits, which aided in the diagnosis of gout in a Vietnamese population. Therefore, we recommend DECT scans for patients with suspected gout and unclear clinical presentation, as well as to monitor short-term and long-term outcomes after treatment. Further prospective studies with a larger sample size are needed to evaluate the effectiveness of this diagnostic method. Moreover, our results suggest the potential benefit of urate-lowering therapy for the treatment of structural joint damage.
